# Rhodamine 19 Alkyl Esters as Effective Antibacterial Agents

**DOI:** 10.3390/ijms25116137

**Published:** 2024-06-02

**Authors:** Pavel A. Nazarov, Vladislav S. Maximov, Alexander M. Firsov, Marina V. Karakozova, Veronika Panfilova, Elena A. Kotova, Maxim V. Skulachev, Yuri N. Antonenko

**Affiliations:** 1 Belozersky Institute of Physico-Chemical Biology, Lomonosov Moscow State University, 119991 Moscow, Russia; amfamf@yandex.ru (A.M.F.); mvk752002@gmail.com (M.V.K.);; 2 Faculty of Bioengineering and Bioinformatics, Lomonosov Moscow State University, 119991 Moscow, Russia; 3 Mitotech LLC, 119991 Moscow, Russia

**Keywords:** MDR pumps, AcrAB-TolC, mitochondria-targeted antioxidants, rhodamine, phosphonium

## Abstract

Mitochondria-targeted antioxidants (MTAs) have been studied quite intensively in recent years as potential therapeutic agents and vectors for the delivery of other active substances to mitochondria and bacteria. Their most studied representatives are MitoQ and SkQ1, with its fluorescent rhodamine analog SkQR1, a decyl ester of rhodamine 19 carrying plastoquinone. In the present work, we observed a pronounced antibacterial action of SkQR1 against Gram-positive bacteria, but virtually no effect on Gram-negative bacteria. The MDR pump AcrAB-TolC, known to expel SkQ1, did not recognize and did not pump out SkQR1 and dodecyl ester of rhodamine 19 (C12R1). Rhodamine 19 butyl (C4R1) and ethyl (C2R1) esters more effectively suppressed the growth of Δ*tolC Escherichia coli*, but lost their potency with the wild-type *E. coli* pumping them out. The mechanism of the antibacterial action of SkQR1 may differ from that of SkQ1. The rhodamine derivatives also proved to be effective antibacterial agents against various Gram-positive species, including *Staphylococcus aureus* and *Mycobacterium smegmatis*. By using fluorescence correlation spectroscopy and fluorescence microscopy, SkQR1 was shown to accumulate in the bacterial membrane. Thus, the presentation of SkQR1 as a fluorescent analogue of SkQ1 and its use for visualization should be performed with caution.

## 1. Introduction

Due to the rapidly developing multidrug resistance, the problem of finding effective antibacterial agents to protect humanity from infectious diseases is extremely urgent. In the present study, we screened derivatives of rhodamine 19, namely esters of this highly fluorescent dye with a variable alkyl chain length, for the antibacterial effect against various species. The fact is that alkyl derivatives of rhodamine 19 are mitochondria-targeted lipophilic cations that can accumulate in energized mitochondria due to the negative charge of their matrix with respect to the cytosol. The rhodamine analogs can cause the dissipation of the membrane potential due to their protonophoric activity [1,2], thus suppressing ATP synthesis. In addition, some of them are inhibitors of mitochondrial F1-ATPase [3,4,5,6]. It is known that bacterial cells are similar in their bioenergetics to mitochondria, and therefore many mitochondria-targeted compounds are also effective against bacteria. A recent publication has demonstrated the benefits of searching for new antibiotics among rhodamine analogs [7]. In addition, several studies revealed effective inhibitors of P-glycoprotein efflux protein among rhodamine derivatives [8,9]. Here, we estimated the antibacterial efficacy of rhodamine 19 alkyl esters, as compared to alkyl derivatives of another lipophilic cation, triphenylphosphonium, and tried to find a pump withdrawing the rhodamine analogs from bacterial cells.

Previously, Skulachev’s group [10,11,12,13] proposed the use of triphenylphosphonium derivatives as mitochondria-targeted substances. This approach was also put forward by Murphy and co-workers [14,15,16], and, to date, many molecules based on triphenylphosphonium derivatives have been created [17], including the most famous MitoQ and SkQ1.

Together with SkQ1 (10-(60-plastoquinonyl) decyltriphenylphosphonium), its fluorescent “analog” SkQR1 (10-(60-plastoquinonyl) decylrhodamine 19) was created [18], which was often used to visualize the distribution of SkQ1 in cell membranes [19]. SkQ1 and SkQR1 are selectively accumulated in mitochondria and have high antioxidant efficiency in living cells and in animal models. SkQR1 and SkQ1 protected cells from apoptosis induced by oxidative stress [20].

Similar to SkQ1, SkQR1 was considered to have no antibacterial properties [21]. Later, we discovered the antibacterial properties of the mitochondria-targeted antioxidants SkQ1 and MitoQ and the mechanism of resistance to them [22,23,24]. On the other hand, it has long been known that Rhodamine 6G (C2R1), like SkQ1, is a substrate of the AcrAB-TolC pump [25,26,27,28,29,30], so there was no doubt that this pump is able to expel SkQR1. Moreover, SkQR1 seems to be pumped out in mammalian cells by the Pgp 170 pump [19], belonging to the ATP-binding cassette (ABC) family.

It should be emphasized that Gram-negative bacteria have other pumps that can recognize and pump out rhodamine derivatives, for example, NorM from *Neisseria gonorrheae* [31], KexD from *Klebsiella pneumoniae* [32], MexHI-OpmD from *Pseudomonas aeruginosa* [33] and *Vibrio parahaemolyticus*, which has a homologue to YdhE in *E. coli,* but differs in substrate specificity [34]. Gram-positive bacteria also have pumps that recognize rhodamines, for example, QacA from *S. aureus* and Bmr from *Bacillus subtilis* [35,36,37]. Moreover, the expression of the *qacA* gene from *S. aureus* in the bacterium *B. subtilis* led to an increase in resistance, which may indicate the existence of some differences in bacterial efflux systems, even in Gram-positive bacteria with similar cell envelope structures [38]. Thus, the history of resistance to rhodamine derivatives is not as simple as in the case of SkQ1, which is pumped solely by the AcrAB-TolC pump under physiological conditions. Despite all this, diversity-oriented rhodamine libraries continue to be considered promising tools for combatting resistant pathogens due to their low inducibility resistance [7]. Of note, novel chemical scaffolds inhibiting the AcrAB-TolC efflux pump are currently studied [39].

The present study is focused on the antibacterial activity of rhodamine 19 alkyl esters, including the rhodamine-derived mitochondria-targeted antioxidant SkQR1, as compared to SkQ1. In particular, although SkQR1 has an antibacterial effect against Gram-positive bacteria, its ability to penetrate the complex membranes of Gram-negative bacteria is significantly inferior to SkQ1.

## 2. Results and Discussion

### 2.1. Antibacterial Activity of Rhodamine Derivatives

According to the data on MICs presented in Table 1, the antibacterial activity of SkQR1 against Gram-positive bacteria *B. subtilis* was comparable to the previously shown antibacterial activity of SkQ1 [10]. Unlike phosphonium derivatives, the activity of rhodamine 19 alkyl esters did not decrease with the shortening of alkyl length, but rather increased, reaching a maximum in the case of rhodamine 6G and C4R1 (Table 1). From our previous work [40] it is known that C12R1 is more effective in reducing membrane potential than C4R1; therefore, the antibacterial effect of SkQR1 and CnR1 does not correlate with a decrease in the membrane potential, as is the case with SkQ1 and CnTPP. It must be noted that all of the above also holds true for *S. aureus* and *M. smegmatis*, which demonstrated MIC of around 1.4–2.8 µg/mL for SkQR1.

SkQ1 is an effective antibacterial agent against both *M. smegmatis* and *Mycobacterium tuberculosis* [22,41]; therefore, we also expected a strong antibacterial effect of the rhodamine derivatives. A greater effect of C4R1 on *M. smegmatis* in comparison with *B. subtilis* may be explained via the differences in rhodamine recognition by MDR pumps (Bmr of *B. subtilis* and LfrA of *M. smegmatis* [42]), easier access due to mycobacterial porins [43,44], or suboptimal levels of pump and porin synthesis [45,46].

**Figure 1:**
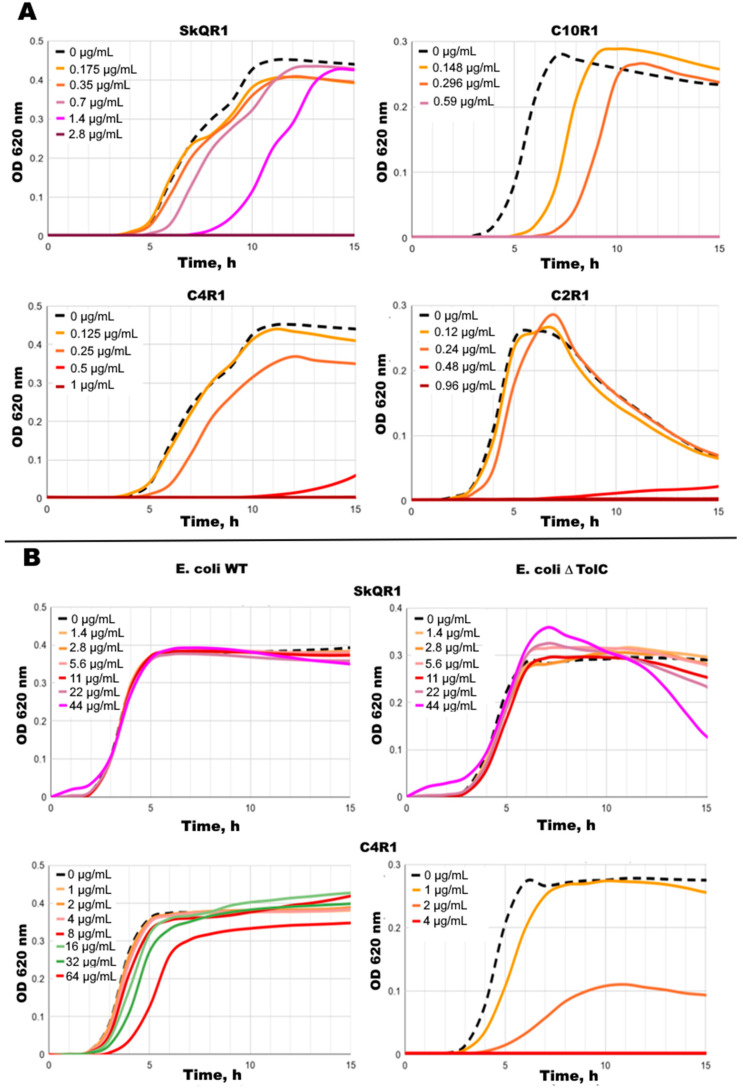
Kinetic growth curves. (**A**) Effect of 0.12–2.8 μg/mL SkQR1 (**top panel, left**), C10R1 (**top panel, right**), C4R1 (**bottom panel, left**), and C2R1 (**bottom panel, right**) on the growth of *B. subtilis*. (**B**) Effect of 1–64 μg/mL SkQR1 (**top panel**), and C4R1 (**bottom panel**) on the growth of *E. coli* WT (**left**) and ∆TolC (**right**). Kinetic growth curves for SkQ1 are presented in Figure S1.

The antibacterial activity of SkQR1 against the Gram-negative bacteria *E. coli* was significantly weaker than that of SkQ1 (Table 1). At the same time, the deletion mutant for the TolC protein of the main MDR pump of *E. coli* AcrAB-TolC did not demonstrate the decreased resistance towards SkQR1, which we observed in the case of SkQ1 [22]. It is known from the literature [25,26,27,28,29,30] that TolC-containing pumps are involved in the extrusion of rhodamine 6G out of the bacterial cells, so it was unexpected to see the resistance in the deletion mutant for the TolC protein with respect to SkQR1 and C12R1. However, C4R1 and C2R1 (Rhodamine 6G) effectively suppressed the growth of the ∆*tolC* strain. At the same time, the resistance to rhodamine 19 alkyl esters in the deletion mutant for the TolC protein increased with increasing the length of the alkyl fragment. Kinetic growth curves also confirmed this observation (Figure 1B).

### 2.2. AcrAB-TolC Transporter Is Responsible for E. coli Resistance to C4R1 but Not to SkQR1

**Figure 2:**
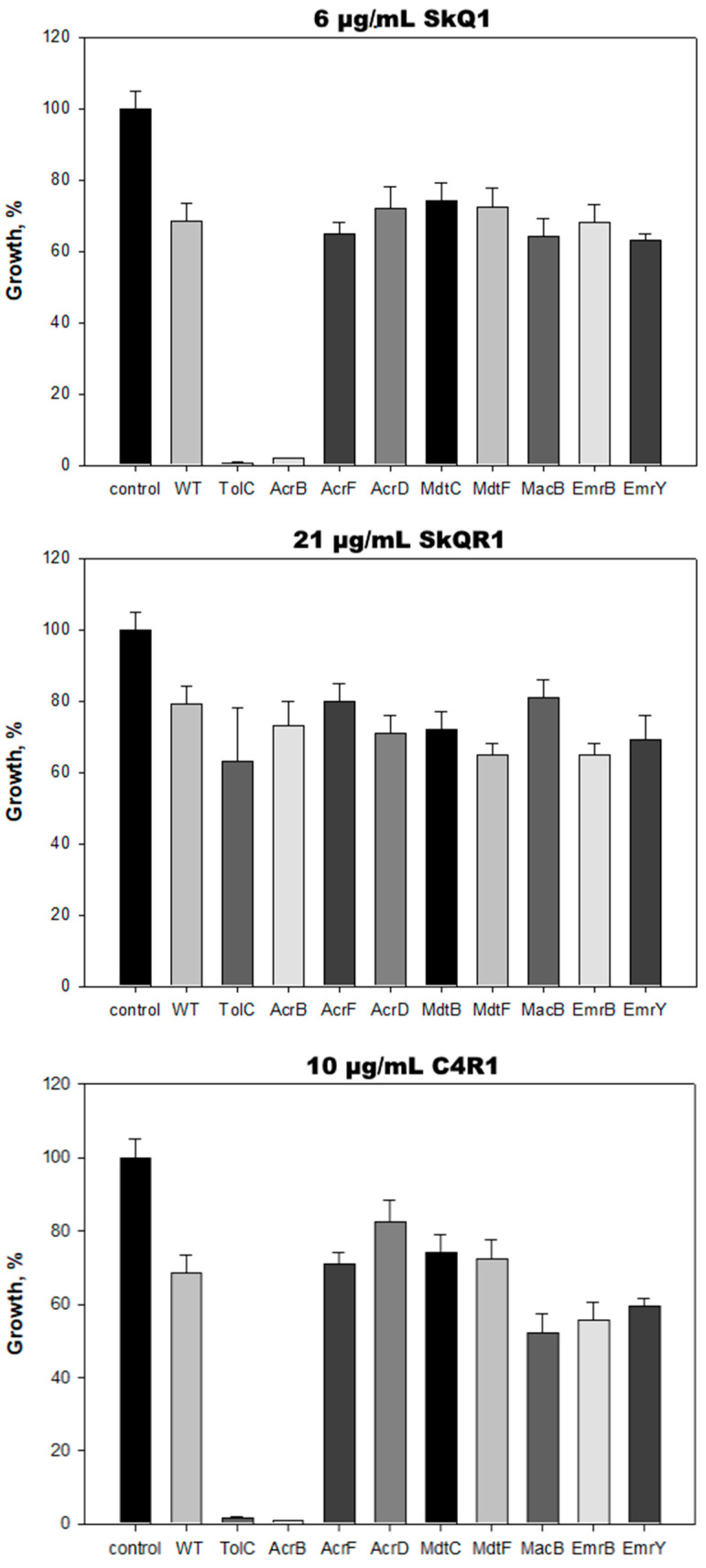
Growth of *E. coli* strains having deletions in various transporters in the presence of 6 μg/mL SkQ1 (**top**), 21 μg/mL SkQR1 (**middle**), and 10 μg/mL C4R1 (**bottom**). Substances were added at “0” time to the LB medium. Growth was evaluated after 15–24 h incubation at 37 °C by absorbance at 620 nm. The growth of WT *E. coli* cells in the absence of substances is referred to as a control, and, in the presence of substances, is referred to as WT. The data points represent mean ± SD of three experiments.

In the Gram-negative bacteria *E. coli*, the TolC protein plays a key role in the multidrug resistance. Based on this protein, at least eight MDR pumps are formed, the main one of which is the AcrAB-TolC pump. Each of them can expel out a wide range of substrates, many of which are removed from the cell by more than one pump [47]. Thus, in *E. coli*, TolC interacts with a variety of inner membrane transporters, thereby enabling the bacteria to expel structurally diverse molecules, such as enterotoxins, antibiotics and antibacterial peptides, bile salts, and some other organic compounds. The Δ*tolC E. coli* mutant demonstrated similar sensitivity to SkQ1 as *B. subtilis*; in particular, 0.6–1.2 μg/mL of SkQ1 completely suppressed the growth of the mutant. Since the Δ*tolC E. coli* mutant did not demonstrate similar sensitivity to SkQR1 as *B. subtilis*, we hypothesized that an increase in the alkyl moiety may lead to the loss of recognition of the molecule by the AcrAB-TolC pump, which was confirmed in the experiment with a series of deletion mutants (Figure 2). None of the TolC-containing pumps were significantly involved in SkQR1 pumping.

In contrast to SkQR1, C4R1 was expelled out of the bacterial cells by TolC-containing pumps. However, the Δ*tolC E. coli* mutant did not demonstrate the same sensitivity to SkQR1 as *B. subtilis*, which indicates the involvement of several pumps in the withdrawal of C4R1, as shown by our experiments with the deletion mutants. We hypothesize that, of the eight TolC-containing *E. coli* pumps (AcrAB-TolC, AcrAD-TolC, AcrEF-TolC, MdtABC-TolC, MdtEF-TolC, EmrAB-TolC, EmrKY-TolC, MacAB-TolC [22]), one or more of the MacAB-TolC, EmrAB-TolC, or EmrKY-TolC pumps might be involved in C4R1 extrusion. However, apparently, the AcrAD-TolC pump is not involved in this process.

### 2.3. Rhodamine Derivatives Are Localized on the Bacterial Membrane

**Figure 3:**
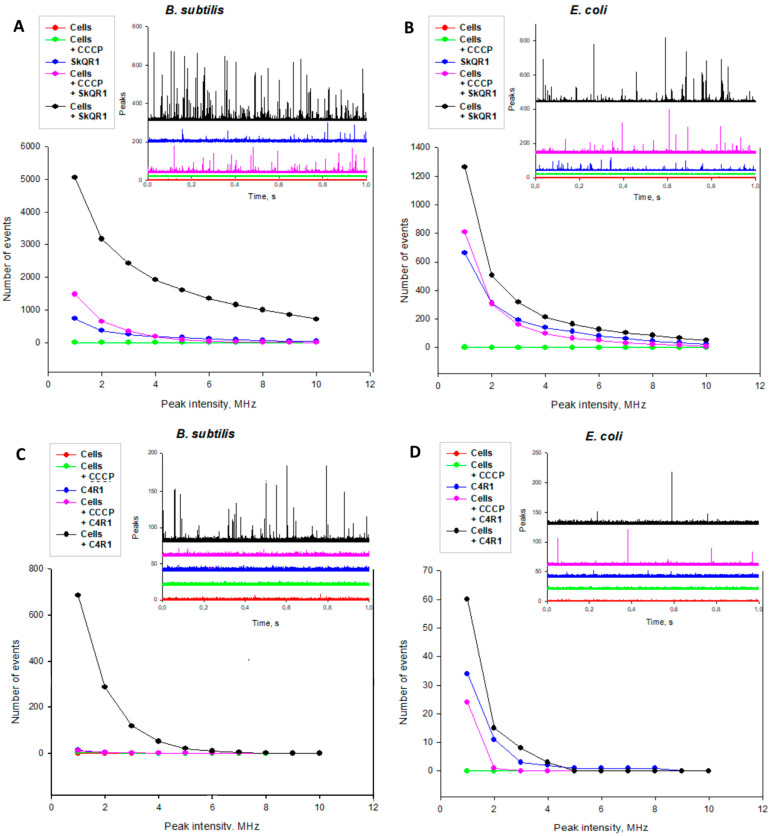
Accumulation of SkQR1 and C4R1 by *B. subtilis* and *E. coli* cells (black) and the effect of CCCP (10 µM) on it (purple), monitored using FCS: (**A**) accumulation of SkQR1 in *B. subtilis* cells, (**B**) accumulation of SkQR1 in *E. coli* cells, (**C**) accumulation of C4R1 in *B. subtilis* cells, (**D**) accumulation of C4R1 in *E. coli* cells. Cells *E. coli* or *B. subtilis* (red), rhodamine derivatives SkQR1 or C4R1 (blue), and cells with added CCCP uncoupler were used as controls. The fluorescence intensity traces of SkQR1 and C4R1 recorded with the FCS set-up in the presence or absence of bacterial cells (10^6^ per mL of PBS). The corresponding dependences of the number of peaks with the fluorescence intensity F exceeding the threshold F_0_, n(F > F_0_), on the value of F_0_.

Wherein, it remains a mystery what the actual mechanism of the antibacterial action of the rhodamine derivatives is. To understand what is happening, it is necessary to evaluate the localization of the rhodamine derivatives in the cell. Fluorescence correlation spectroscopy (FCS) measures fluctuations in the emission signal of a small number of fluorescent molecules diffusing into and out of the confocal volume of an excitation laser (Figure 3). Solutions of the rhodamine derivatives without bacterial cells emitted the fluorescence signal with low-amplitude fluctuations because there were a large number of free rhodamine molecules in the confocal volume.

In the case of the incubation of *B. subtilis* cells in the presence of SkQR1 or C4R1, the fluorescence recording contained peaks of a rather high amplitude, which corresponded to the appearance of cells bearing a large number of rhodamine molecules. Moreover, the number of peaks in the case of SkQR1 was approximately an order of magnitude greater than in the case of C4R1. Thus, the more hydrophobic SkQR1 was more effectively accumulated in cells.

Upon the addition of the classical uncoupler CCCP, a sharp decrease in the binding of the rhodamine derivatives to cells was observed, which indicates the voltage-dependent accumulation of rhodamine molecules in *B. subtilis* cells.

In the case of the incubation of *E. coli* cells in the presence of SkQR1 or C4R1, the fluorescence recording also contained peaks of rather high amplitude, which corresponded to the appearance of cells bearing a large number of rhodamine molecules. When comparing the number of peaks caused by the addition of SkQR1 and C4R1, as in the case of *B. subtilis*, we saw a difference in cell binding. Thus, the number of peaks in the case of SkQR1 was approximately an order of magnitude greater than in the case of C4R1, which also indicates that the more lipophilic SkQR1 accumulated more strongly in cell membranes.

Moreover, the numbers of both SkQR1 and C4R1 peaks in *E. coli* and in *B. subtilis* differed substantially as follows: in the case of *E. coli*, there were noticeably fewer of them (~1300 vs. 5100 in the case of SkQR1 and ~60 vs. 700 in the case of C4R1).

**Figure 4:**
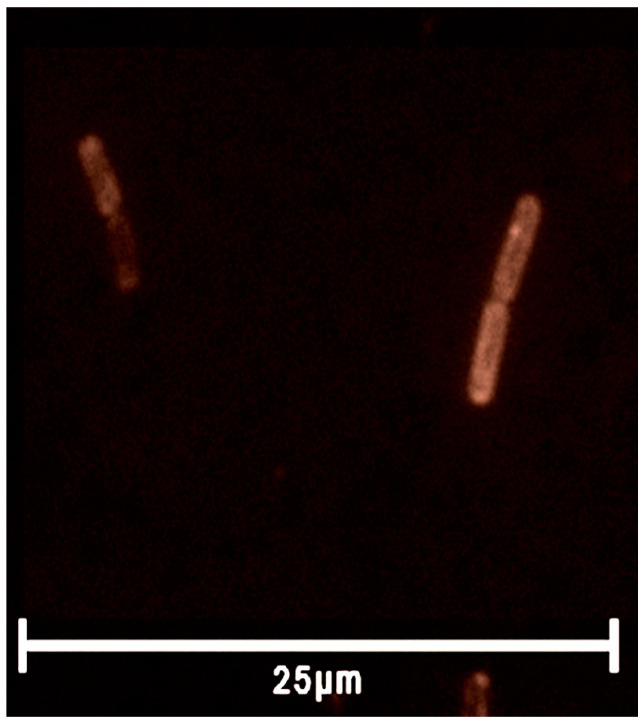
Fluorescence from *B. subtilis* cells stained with SkQR1. The cells were incubated with 3.5 µg/mL of the dyes for 5 min, washed with LB, and imaged.

To confirm our findings, we used fluorescence microscopy, enabling one to visualize SkQR1 on the bacterial cell membrane (Figure 4). Obviously, we saw bright staining along the perimeter of the cell and a duller area in the central part of the bacterium, which indicates that SkQR1 is specifically localized on the bacterial membrane. Based on the above results, it can be concluded that SkQR1 adsorbed to *B. subtilis* cells much more effectively than C4R1, while the antibacterial effect of SkQR1 on *B. subtilis* was somewhat less than that of C4R1. Moreover, both compounds bound well to *E. coli*, but were practically nontoxic to them. One should distinguish between the binding of SkQR1 or C4R1 to the cell membrane and the presence of the compounds in cytoplasm. Unlike C4R1, SkQR1 is expected to predominantly localize in the membranes due to its much higher lipophilicity when compared to C4R1. Taken together, our data indicate that the preferential localization of the rhodamine analogs in the membrane markedly attenuated their antibacterial effect. Although at the moment, the target of rhodamines’ action in the cytoplasm is unclear, we can refer to our work on the effect of a series of rhodamine 19 derivatives on the functioning of the alpha-hemolysin nanopore [48]. Among the rhodamine 19 alkyl esters, channel blocking was observed specifically with the hydrophilic analogs (C4R1 and C2R1), while the more hydrophobic rhodamines were much less effective. In other words, C4R1 and C2R1 interacted stronger with the hydrophilic interior of the channel when compared to longer alkyl chain derivatives.

## 3. Materials and Methods

### 3.1. Materials

**Figure 5:**
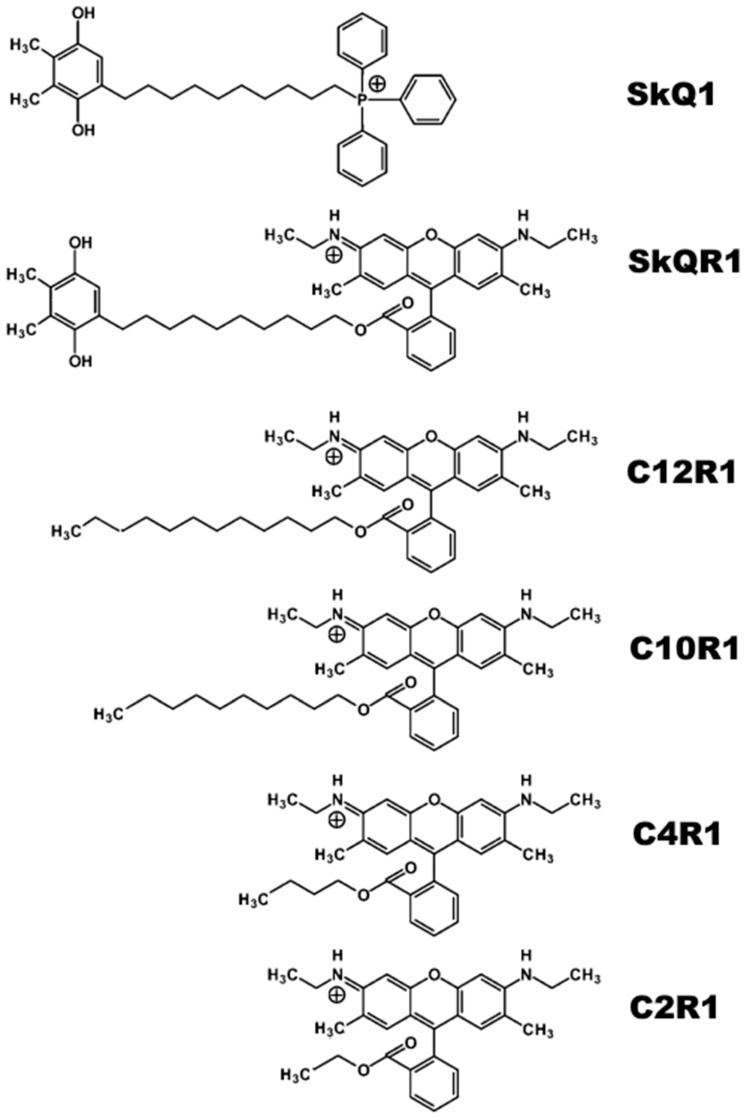
Chemical structures of SkQ1, SkQR1, and alkyl ester derivatives of rhodamine 19 (CnR1).

Components of the bacterial Luria-Bertani (LB) media were purchased from Helicon Company (Moscow, Russia), and the Becton Dickinson (Franklin Lakes, NJ, USA) Mueller-Hinton (MH) medium was purchased from HiMedia Laboratories (Mumbai, India). Other reagents were from Sigma-Aldrich (St. Louis, MO, USA). Derivatives of rhodamine (Figure 5) were synthesized by Galina A. Korshunova and Natalia V. Sumbatyan, as described in [18].

### 3.2. Bacterial Strains

The standard laboratory strains used in the study included Bacillus subtilis subsp. subtilis Cohn 1872 strain BR151 (trpC2 lys-3 metB10) and Escherichia coli Castellani and Chalmers 1919 strain MG1655 (F-, ilvG, rfb-50 rph-1). Additionally, Staphylococcus aureus (No. 144) and Mycobacterium smegmatis (No. 377) were obtained from the microorganism collection at Lomonosov Moscow State University.

Deletion strains used in the study included ECK3026 (lacking the tolC gene), ECK0456 (lacking the acrB gene), ECK2465 (lacking the acrD gene), ECK3253 (lacking the acrF gene), ECK2071 (lacking the mdtB gene), ECK3498 (lacking the mdtF gene), ECK0870 (lacking the macB gene), ECK2363 (lacking the emrY gene), and ECK2680 (lacking the emrB gene). These deletion strains were generously provided by Dr. H. Niki from the National Institute of Genetics in Japan.

The bacterial cells were cultured at either 30 °C or 37 °C in Luria-Bertani (LB) or Mueller-Hinton (MH) medium with agitation at 140 rotations per minute (rpm).

### 3.3. Growth Suppression Assay and MIC Determination

The growth suppression assay was performed through inoculating 200 µL of bacterial cultures 5 (~5 × −10 cells/mL) into 96-well plates (Eppendorf AG, Hamburg, Germany). The compounds were diluted in a 96-well microtiter plate to final concentrations ranging from 0.24 to 89.6 µg/mL of C_n_R1, SkQR1, or SkQ1 in a 250-mL aliquot of the total volume. The bacteria were allowed to grow for 18 or 21 h at 30 °C or 37 °C. MICs, the lowest concentrations that completely inhibited the bacterial growth, were determined using Mueller-Hinton broth microdilution, as recommended by CLSI, using in-house-prepared panels. Bacterial growth was observed visually alongside CFU and OD measurements [49]. Optical densities at 620 nm were obtained using a Thermo ScientificMultiskan FC plate reader with an incubator (Thermo Fisher Scientific, Waltham, MA, USA). Experiments were carried out in triplicate.

### 3.4. Fluorescence Microscopy

#### 3.4.1. Agarose Pads Preparation

A mixture of 2% low-melting agarose in medium was heated to boiling and subsequently cooled to 42 °C. Using this agarose solution, an agarose pad was created by pouring 1 mL of the mixture onto a 22 × 22 mm cover glass and placing another cover glass of the same size on top. The pad was left undisturbed to solidify for 45 min, following which one cover glass was removed. The solidified agarose pad was then sliced into smaller individual pads using a scalpel, resulting in two pads for each strain [50].

#### 3.4.2. Sample Pads Preparation

Overnight bacterial cell cultures were thinned by a factor of 10 using fresh LB media. The bacterial cell suspensions were then treated with 3.5 µg/mL SkQR1 for 5 min, and 5–7 µL were applied to the top of the pads. Once dried (around 15 min), the pads were flipped and moved to a 35 mm confocal glass bottom dish SPL 100350 (SPL Life Sciences Co., Pocheon-si, Republic of Korea) for imaging.

#### 3.4.3. Equipment Setup

In order to examine the distribution of SkQR1 in *B. subtilis* cells, we employed a configuration consisting of the inverted motorized BZ-9000 BioRevo fluorescence microscope from Keyence (Itasca, IL, USA) fitted with an HC PL Apo 100 × 1.40 oil lens manufactured by Nikon in Tokyo, Japan. The setup also included a TRITC filter cube with an excitation filter of 545/25 and an emission filter of 605/70, as well as a temperature control chamber. The images obtained were processed using the FIJI ImageJ software tools (version 1.52u) [51,52].

### 3.5. Fluorescence Correlation Spectroscopy

Fluorescence correlation spectroscopy (FCS) measurements were conducted using a custom-built FCS setup [53] that included an Olympus IMT-2 inverted microscope equipped with a 40×, NA 1.2 water immersion objective from Carl Zeiss, Jena, Germany. A Nd:YAG solid-state laser served as the excitation source. The fluorescence that passed through a suitable dichroic beam splitter and a long-pass filter was directed onto a 50 µm core fiber connected to an avalanche photodiode from PerkinElmer Optoelectronics in Fremont, CA, USA. The signal was then correlated using a correlator card from Correlator.com in Bridgewater, NJ, USA. Data acquisition was performed over a 30 s period. The experimental data were acquired under stirring conditions, which significantly increased the number of observed events, improving the method’s resolution by three orders of magnitude. Peak intensities of the fluorescence traces with a sampling interval of 25 µs were analyzed utilizing the WinEDR Strathclyde Electrophysiology Software (version 2.6.9), developed by J. Dempster at the University of Strathclyde, UK. This software, initially intended for the single-channel analysis of electrophysiological data, enabled the quantification of the number of peaks [n(F>F0)] in the FCS signal that exceeded a defined threshold value F0. Additionally, a custom program with a similar algorithm, named Saligat (available upon request), was also utilized.

### 3.6. Deletion Mutants of TolC-Requiring Transporters Growth Suppression Screening

A panel of *E. coli* deletion mutants [22,23] was chosen. The selected bacterial strains from the panel were diluted in fresh LB media after overnight incubation. Furthermore, 200 µL of bacterial cell cultures at a concentration of 5 × 10^5^ cells/mL were added to 96-well plates. Different concentrations of preselected SkQ1, SkQR1, and C4R1 (6 µg/mL, 21 µg/mL, and 10 µg/mL, respectively) were then introduced to each mutant, and the optical density at 620 nm was measured using a Thermo Scientific Multiskan FC plate reader. The cells were allowed to grow for 21 h at 37 °C, and the optical density at 620 nm was measured again. All experiments were conducted in triplicate at minimum.

## 4. Conclusions

Mitochondrial-targeted antioxidants are promising antibacterial agents whose mechanism of action is still surrounded by mystery and apparent cytotoxicity problems. Some features are already becoming clear, for example, the role of MDR pumps in resistance not only to antibiotics, but to substances that belong to protonophores [22], emphasizing the connection between bioenergetics, MDR pumps and antibiotic resistance [54,55,56,57].

The mechanisms of action of antibacterial compounds can be divided into six classes as follows: (1) the inhibition of cell wall biosynthesis; (2) the disruption of nucleic acid synthesis; (3) the disruption of protein synthesis; (4) the disruption of the integrity of the plasma membrane; (5) the negative modulation or blocking of key metabolic pathways; and (6) the disruption of energy generation in the form of ATP or H+/Na+ gradient [47,58,59,60]. Although SkQR1, like SkQ1, reduces the membrane potential due to the protonophore cycle on the membrane and thus can be classified as an antibacterial substance that disrupts energy generation in the bacterial cell, our data indicate the existence of another mechanism of SkQR1 action on bacteria.

In conclusion, we can say that the results allow us to take a new look at the general problem of the apparent toxicity of such compounds. Resolving toxicity problems is urgent, as rhodamine derivatives, especially triphenylphosphonium derivatives, are also vectors for the delivery of other drugs into mitochondria and bacteria [17,61,62,63,64,65,66,67,68,69,70].

Another problem highlighted in this article is the recognition of substances by MDR pumps [71,72,73,74,75,76]. This is especially important in light of the work of the main MDR pump of Gram-negative bacteria AcrAB-TolC and its structural analogues [24]. Obviously, by varying the length of the linker, we can change the pumps’ recognition of their substrates, which is important for modern pharmacology and the creation of new antibacterial substances.

Of course, it is necessary to understand that the concept of SkQR1 as a fluorescent analogue of SkQ1 was strongly simplified. Apparently, the mechanism of action of SkQR1 differs from that of SkQ1, which determines their different properties. Therefore, SkQR1 should be used with caution as a fluorescent analogue of SkQ1.

## Figures and Tables

**Table 1:** Suppression of the growth of bacteria by Rhodamine 19 derivatives. Values of minimal inhibitory concentration (MIC, µg/mL) are shown. The MIC for each compound was determined in triplicate in two independent sets.

	*Bacillus* *subtilis*	*Escherichia* *coli*	*Escherichia* *coli* Δ*tolC*	*Staphylococcus* *aureus*	*Mycobacterium* *smegmatis*
SkQ1	0.6–1.2	21	1.2	0.6–1.2	0.6
SkQR1	2.8	89.6<	89.6	2.8	1.4
C12R1	2.4	76<	76	2.4	1.2
C10R1	1.2	75<	38	1.2	1.2
C4R1	0.5–1	64<	4	0.5	0.125
C2R1	0.48–0.96	61<	3.8	0.96	0.48

## Data Availability

Data are contained within the article and Supplementary Materials.

## Supplementary Materials

The following supporting information can be downloaded at https://www.mdpi.com/article/10.3390/ijms25116137/s1.
