# Supplementary material for: Rhodamine 19 Alkyl Esters as Effective Antibacterial Agents
Source: Int J Mol Sci. 2024 Jun 2;25(11):6137. doi: 10.3390/ijms25116137 (PMC11173286; doi:10.3390/ijms25116137)
Supplement: Supplementary file 1 [file ijms-25-06137-s001.zip › ijms-3003006-supplementary.pdf]

International Journal of
Molecular Sciences

MDPI

Supplementary Materials

Rhodamine 19 Alkyl Esters as Effective Antibacterial Agents

Pavel A. Nazarov [1,*], Vladislav S. Maximov [2], Alexander M. Firsov [1], Marina V. Karakozova [1], Veronika Panfilova [1], Elena A. Kotova [1], Maxim V. Skulachev [1,3] and Yuri N. Antonenko [1]

[1] Belozersky Institute of Physico-Chemical Biology, Lomonosov Moscow State University, 119991 Moscow, Russia; amfamf@yandex.ru (A.M.F.); mvk752002@gmail.com (M.V.K.); verapanfilovaaa@gmail.com (V.P.); kotova@genebee.msu.ru (E.A.K.); max@mitotech.ru (M.V.S.); antonen@genebee.msu.ru (Y.N.A.)
[2] Faculty of Bioengineering and Bioinformatics, Lomonosov Moscow State University, 119991 Moscow, Russia; maximovvlad97@gmail.com
[3] Mitotech LLC, 119991 Moscow, Russia
* Correspondence: nazarovpa@gmail.com

Table of Contents:

1. **Kinetic growth curves for SkQ1**

   Effect of 0.25-1 μM SkQ1 on the growth of bacteria *B. subtilis*…………**S1A**

   Effect of 1-50 μM SkQ1 on the growth of bacteria *E. coli* WT…..………**S1B**

   Effect of 0.1-1.25 μM SkQ1 on the growth of bacteria *E. coli* ΔtolC …...**S1C**

2. **Table of molecular weights for CnR1, SkQR1 and SkQ1**

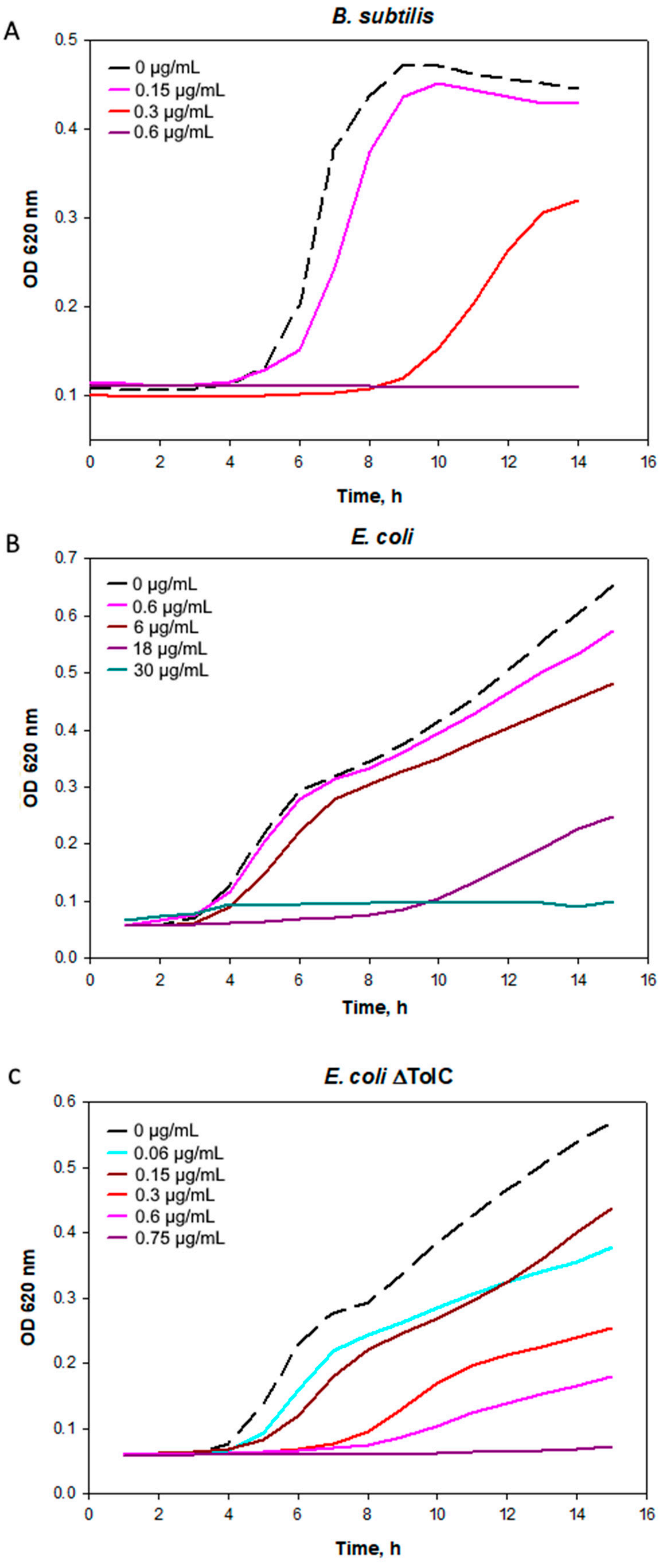

Figure S1. Kinetic growth curves for SkQ1 (**A**) Effect of 0.15-0.6 µg/mL SkQ1 on the growth of *B. subtilis*. (**B**) Effect of 0.6-30 µg/mL SkQ1 on the growth of *E. coli* WT. Effect of 0.06-0.75 µg/mL SkQ1 on the growth of *E. coli* ΔTolC.

Table 1S. Table of molecular weights for CnR1, SkQR1 and SkQ1.

	Molecular formula	*Molecular weight*
**SkQ1**	$C_{36}H_{42}BrO_2P$	617.6 g/mol
**SkQR1**	$C_{44}H_{56}N_2O_6$	708.9 g/mol
**C12R1**	$C_{38}H_{47}N_2O_3$	619 g/mol
**C10R1**	$C_{36}H_{43}N_2O_3$	591 g/mol
**C4R1**	$C_{30}H_{35}N_2O_3$	507 g/mol
**C2R1**	$C_{28}H_{31}N_2O_3$	479 g/mol
